# International ResearchKit App for Women with Menstrual Pain: Development, Access, and Engagement

**DOI:** 10.2196/14661

**Published:** 2020-02-11

**Authors:** Jiani Wang, Alizé A Rogge, Mike Armour, Caroline A Smith, Christopher R D’Adamo, Claudia R Pischke, Hung-Rong Yen, Mei-Yao Wu, Ari Ojeda Ocampo Moré, Claudia M Witt, Daniel Pach

**Affiliations:** 1 Charité – Universitätsmedizin Berlin, corporate member of Freie Universität Berlin, Humboldt-Universität zu Berlin, and Berlin Institute of Health, Institute for Social Medicine, Epidemiology and Health Economics Berlin Germany; 2 NICM Health Research Institute Western Sydney University Sydney Australia; 3 Center for Integrative Medicine School of Medicine University of Maryland Baltimore, MD United States; 4 Institute of Medical Sociology, Centre for Health and Society, Medical Faculty Heinrich Heine University Düsseldorf Düsseldorf Germany; 5 School of Chinese Medicine China Medical University Taichung Taiwan; 6 Department of Chinese Medicine China Medical University Hospital Taichung Taiwan; 7 Integrative Medicine and Acupuncture Division University Hospital Federal University of Santa Catarina Florianópolis, Santa Catarina Brazil; 8 Institute for Complementary and Integrative Medicine University Zurich and University Hospital Zurich Zurich Switzerland

**Keywords:** dysmenorrhea, mHealth, mobile applications, acupressure, pain, behavior change techniques (BCTs), ResearchKit, recruitment

## Abstract

**Background:**

Primary dysmenorrhea is a common condition in women of reproductive age. A previous app-based study undertaken by our group demonstrated that a smartphone app supporting self-acupressure introduced by a health care professional can reduce menstrual pain.

**Objective:**

This study aims to evaluate whether a specific smartphone app is effective in reducing menstrual pain in 18- to 34-year-old women with primary dysmenorrhea in a self-care setting. One group of women has access to the full-featured study app and will be compared with 2 control groups who have access to fewer app features. Here, we report the trial design, app development, user access, and engagement.

**Methods:**

On the basis of the practical implications of the previous app-based study, we revised and reengineered the study app and included the ResearchKit (Apple Inc) framework. Behavior change techniques (BCTs) were implemented in the app and validated by expert ratings. User access was estimated by assessing recruitment progress over time. User evolution and baseline survey respondent rate were assessed to evaluate user engagement.

**Results:**

The development of the study app for a 3-armed randomized controlled trial required a multidisciplinary team. The app is accessible for the target population free of charge via the Apple App Store. In Germany, within 9 months, the app was downloaded 1458 times and 328 study participants were recruited using it without external advertising. A total of 98.27% (5157/5248) of the app-based baseline questions were answered. The correct classification of BCTs used in the app required psychological expertise.

**Conclusions:**

Conducting an innovative app study requires multidisciplinary effort. Easy access and engagement with such an app can be achieved by recruitment via the App Store. Future research is needed to investigate the determinants of user engagement, optimal BCT application, and potential clinical and self-care scenarios for app use.

**Trial Registration:**

ClinicalTrials.gov NCT03432611; https://clinicaltrials.gov/ct2/show/NCT03432611 (Archived by WebCite at http://www.webcitation.org/75LLAcnCQ).

## Introduction

### Background

In recent years, increasing smartphone access has enabled the advancement and widespread use of smartphone apps [1,2]. Apps are a promising tool for people with a wide variety of health conditions and may be particularly useful to guide and support individuals in the self-management of these conditions [3,4]. A recent systematic review on apps in pain management concluded that apps might be beneficial for patients, particularly in an outpatient setting, but that there is a need for more scientific knowledge [5]. Furthermore, in an Australian national survey on mobile health (mHealth) in women with polycystic ovary syndrome [6], current evidence-based information was considered to be the most desirable app feature. Thus, an app with evidence-based information on menstrual pain might be of great value for patients suffering from this common problem.

Menstrual disorders are highly prevalent among women of reproductive age, and especially in young women; they commonly include period pain and mood disturbances [7]. Primary dysmenorrhea is defined as menstrual pain in the absence of underlying pathology, with the pain commonly starting within 3 years of menarche (the first menstrual period) [8]. A characteristic symptom of primary dysmenorrhea is crampy, colicky spasms of pain below the belly button, occurring within 8 to 72 hours of menstruation and peaking within the first few days as menstrual flow increases [9]. Many women with dysmenorrhea also experience other menstrual-related symptoms such as back pain, headaches, bowel changes, nausea, and vomiting [9]. Primary dysmenorrhea has significant negative impacts on education [7] and productivity at work [10]. Current menstrual health literacy and understanding of effective self-care strategies for menstrual symptoms are often poor [11].

In a previous randomized pragmatic trial (trial registration: ClinicalTrials.gov NCT01582724) [12] for women with menstrual pain, a total of 221 women were randomly assigned to 1 of 2 study groups. Both groups received the study app and a short introduction by a health care professional. Although the intervention group had access to acupressure-based features, including visual and written instructions on how to apply self-acupressure before and during menstruation, the control group did not. In addition, the app could send regular reminders to start the acupressure or to fill in questions. For both groups, the app was used to collect the study-related data and support the management of the menstrual period with a simplistic period calendar. Users in the self-acupressure group reported a significant reduction in the mean pain intensity and reported less pain medication intake in comparison with the usual care control group. In addition, two-thirds of the women still used the app and continued to apply self-acupressure after 6 months [12]. Owing to the fast-developing mHealth technology, it was difficult to keep these noteworthy study results relevant for actual implementation. This was, in part, because of the user experience and because the underlying technology soon became outdated. Therefore, a complete modernization and reengineering of the app and the development of a new corresponding trial that examines its effect over a longer duration than undertaken in the initial trial were necessary.

In 2015, Apple Inc introduced ResearchKit as an open-source framework to support clinical researchers conducting structured mobile app–based health studies [1]. This free and reusable framework can simplify the integration of patient recruitment, the consent process, and the data collection in an mHealth study app. A modernization and reengineering of the previous study app using the ResearchKit framework, new software tools, and design guidelines for broader functionalities and an up-to-date interface would allow to verify the study results from our previous trial on a larger scale and in a real-life self-care setting in several different countries across the world. To our knowledge, no ResearchKit app-based interventional studies have been previously conducted targeting women with menstrual pain. By implementing this ResearchKit app, it would be possible to improve self-care for menstrual pain by encouraging users to change their behavior and regularly apply self-care activities, such as exercise, yoga, or self-acupressure.

Michie et al defined the smallest, observable, replicable intervention component with the potential to bring about change in behavior as behavior change techniques (BCTs) [13]. BCTs have been widely applied in electronic health interventions. A prior ResearchKit app-based observational study evaluated the decision making in patients with acute anterior cruciate ligament ruptures [14] and suggested that it might be possible to maintain users’ motivation by providing instant feedback and relevant treatment information. In another study aimed at reducing alcohol consumption via an app [15], self-monitoring, goal setting, action planning, and feedback in relation to goals were identified as BCTs with the greatest potential to reduce alcohol use. A review on apps targeting persons with poor control of type 2 diabetes mellitus also suggests that the majority of BCTs employed are those for the promotion of self-regulatory behavior [16]. However, there is a lack of data for expert validation of BCTs implemented in apps for menstrual pain.

From the recruitment perspective, previous ResearchKit-based studies predominantly used Web-based recruitment. Web-based recruitment has the potential advantage of reaching a broader population quickly, whereas conventional recruitment is usually time consuming and costly. However, the broad reach can potentially bring in people who are not the target population of a particular mHealth study [17]. In an interventional ResearchKit study, enrollment before eligibility screening (number of App Store visits and downloads) and after baseline questions are indicators for user engagement. However, this important measurement has not been widely reported in previous mHealth studies yet.

### Objectives

To address the questions raised above and to gain a greater understanding for conducting mHealth trials, we report the development, user access, and user engagement of our ResearchKit-based study app for an ongoing pragmatic randomized controlled trial (RCT) [18] on menstrual pain.

## Methods

### Study App and Study Design

#### Technical Development of the Study App

The development of the app was started with the aim to modernize the design and technology of the study app *AKUD* (2012-2015) for a new 3-armed study in a self-recruitment self-care setting.

The study app *Luna.* (Luna, period) was developed in a collaborative project by the Institute of Complementary and Integrative Medicine of the University of Zurich, Switzerland, the Institute for Social Medicine, Epidemiology and Health Economics, Charité – Universitätsmedizin Berlin, Germany, and Smart Mobile Factory, Berlin, Germany, based on Apple’s ResearchKit modular concept. The app was coded in Swift 4 with initial full support for English and German and prepared for easy deployment of other languages, such as simplified and traditional Chinese. The design followed the iOS Human Interface Guidelines (2017) and targets young women. The team involved in the development included iOS and back-end developers, designers, medical doctors, public health researchers, psychologists, and experts on integrative medicine and health.

#### Behavior Change Techniques in the Study App

The development of user interaction and feedback wording was based on the previous app. However, during the development of the new app, we used the BCT taxonomy (BCTTv1), according to Michie et al [19], to document BCTs employed in the app. For example, the BCT *goal setting* was implemented to promote the goal of completing certain self-care activities. In addition, bar charts that recorded change in pain and activities were set up based on the BCT *self-monitoring*. The app was developed in English. During the adaption to German and Chinese, the content of the app was always translated with care to ensure that the respective underlying BCTs were not affected.

For the scientific description of an mHealth intervention, a proper description of BCTs implemented in the app is important. For this, expert validation is essential. At a later stage after the app development was completed, 2 psychologists who were not part of the development team independently rated the individual app features to validate the proper use of BCTs according to the BCTTv1 [19]. We compared the list of BCTs (that were intended to be implemented in the app) of 1 app development team member with the rating results of these 2 psychologists. Where there was disagreement regarding which BCT was used in the app, a final agreement was reached in a consensus meeting between the 3 raters (Table 1).

**Table 1:** App features and corresponding behavior change techniques implemented.

App features	Wording and app content	BCTs^a^ (rating)
Introduction to baseline survey	“Hello! To get to know you better, we would like to ask you some more questions. All of your data will be kept strictly confidential and anonymous.”	No BCTs
Baseline survey finished	“Thank you for your patience. Now we have all the necessary baseline information. You can start with the study.”	No BCTs
Notification of doing interventions/fulfilling surveys	“Time to do some activities for your period pain and record your progress.”	Prompts/cues (7.1)
When a survey has been finished	“Well Done!”	Social reward (10.4)
In-app reminder of finishing survey during task days	“Missing Answers. Keep going with the questions, this can help you see your progress.”	Prompts/cues (7.1)
In-app reminder for acupressure	“Apply acupressure. On days where you have pain, we recommend at least twice a day.”	Prompts/cues (7.1)
When the timer for acupressure finished (for all 6 points)	“Well Done! Keep on taking care of yourself.”	Social reward (10.4)
Guide for nontask days	“New questions will appear five days before your next period.”	Prompts/cues (7.1)
Instructions of when to apply acupressure	When to Apply Acupressure. Instructions of when to apply acupressure (time, frequency).	Goal setting (behavior) (1.1); action planning (1.4)
Instructions of how to apply acupressure	How to Apply Acupressure. Instructions of how to apply acupressure (position, strength, and feeling).	Instructions on how to perform a behavior (4.1)
An image and location for each acupressure point	Image and description of locations of acupressure 3 points: spleen 6, liver 3, large intestine 4.	Instructions on how to perform a behavior (4.1); demonstration of behavior (6.1)
Instruction video for self-acupressure	An instruction animation for self-acupressure on 3 points: spleen 6, liver 3, large intestine 4.	Instructions on how to perform a behavior (4.1); demonstration of behavior (6.1)
Self-care recommendation	“Evidence-based information with references of 5 self-care recommendations: exercises; dietary supplementations; heating pad/hot water bottle; yoga; medication.”	Information about health consequences (5.1); credible source (9.1)
Timer for self-acupressure: 1 minute for each point	A counting down timer with a picture of the corresponding acupressure point.	Goal setting (behavior) (1.1); instructions on how to perform a behavior (4.1); demonstration of behavior (6.1)
Dashboard screen	Dashboard screen, including period calendar, diagrams, and charts reviewing pain and survey questions, and a function button for period start/end.	Feedback on behavior (2.2); self-monitoring of behavior (2.3); self-monitoring of outcome(s) of behavior (2.4); feedback on outcome(s) of behavior (2.7)
Journal screen: calendar	Journal screen in calendar view, including period calendar that also displays the completion of survey questions.	Prompts/cues (7.1)
Journal screen: questions	Journal screen in questions view, including a list of survey questions with the date.	No BCTs
Self-care screen	Self-care screen, including a list and icon images for 5 self-care recommendations.	No BCTs

^a^BCT: behavior change technique.

#### Privacy and Data Security

Privacy and data security were considered high priorities during app development. User data collected by the app are encrypted and transferred anonymously. We adhere to the principle of data minimization [20] and collect only data that are absolutely necessary to answer the research questions. Personally identifiable information (PII), such as the name and signature collected during the informed consent procedure provided by Apple ResearchKit, is stored only on the user’s iPhone and will not be sent to the back end. The individual person owning the iPhone (the study participant) will not be identifiable by the data transferred to the study server. A token will be created as an identifier to label the individual study data. Moreover, an app passcode is implemented to avoid unintended access to the app. Collection of information by the app can be stopped at any time by withdrawing from the study, using a specific button in the app’s settings, and uninstalling the app. Data will be collected anonymously. In addition, the study team of the coordinating office in Germany is supervised by the data protection officer of the Charité—Universitätsmedizin Berlin. The other participating centers are supervised by their respective institutions.

#### Study Design

We will conduct a 3-armed, randomized pragmatic trial [18,21] to evaluate whether the smartphone app is effective in reducing menstrual pain in 18- to 34-year-old women with primary dysmenorrhea. We will compare the group of women who has access to the full-featured study app with 2 control groups who have access to fewer app features. After within-app verification of eligibility for the study, eligible women will be randomly allocated to one of the 3 groups in a 1:1:1 ratio. The potential group allocations are as follows: full-featured app version (self-care information + self-acupressure feature), control intervention I (only self-care information feature), or control intervention II (only self-acupressure feature). The app contains the interventions for all 3 groups, but the content is only unlocked and presented to the user depending on their group allocation. Study participants can use the app for the whole study duration of 12 menstruation cycles. The primary outcome of the study is the mean pain intensity measured with the in-app numerical rating scale (NRS) ranging from 0, *no pain*, to 10, *most intense pain imaginable*, on the painful days during the sixth menstruation after starting the intervention (approximately 6 months from trial start depending on cycle length). It will be calculated by adding up the daily values from the start of the menstruation until the end of bleeding and then dividing them by the number of days with available values. NRS is a common measure of pain intensity that has been utilized in many previous studies [22-24], including studies of menstrual pain [25,26]. Secondary outcome measures are described in more detail on ClinicalTrials.gov (NCT03432611).

The decisions on study design of this trial are based, in part, on decisions of the stakeholder advisory group from the corresponding previous trial and its results [12]. As no member of the study team was specialized in gynecology, this expertise was represented by a gynecologist appointed to the advisory group. Our stakeholder advisory group included a female gynecologist, a 16-year-old woman with dysmenorrhea, a female teacher, 2 acupuncture experts, and a mind-body medicine expert [27].

#### Intervention Components

Furthermore, 5 days before the anticipated start of the menstruation until the end of bleeding, notifications from the app will remind all the groups of participating women to complete questions and perform self-care activities, such as self-acupressure or yoga, depending on the group allocation.

The self-care feature will offer information on self-care for menstrual pain, including evidence-based information about exercise, nutrition and dietary supplementation, heating pad/hot water bottle, yoga, and when to consult a doctor and regarding how primary dysmenorrhea is treated in most cases (see Multimedia Appendix 1).

The acupressure feature will offer detailed written and multimedia descriptions of the acupressure to be used for menstrual pain (see Multimedia Appendix 2). A total of 3 acupressure points will be described that should be massaged bilaterally, if possible, twice a day, up to 5 times per day, starting from 5 days before menstruation until the end of menstruation. Each point should be massaged for 1 min (ie, altogether 6 min should be spent for 1 acupressure session). A visual timer for the acupressure will indicate desirable length of acupressure. In addition, an in-app notification on the app’s dashboard will remind users to practice acupressure during painful days at least twice daily.

The acupressure intervention resulted from a written consensus process with international acupuncture experts from China, Germany, and the United States of America [27] and was already evaluated in an RCT previously conducted by our group demonstrating effectiveness of the intervention [12]. The acupuncture points SP6 (Sanyinjiao), LI4 (Hegu), and LR3 (Taichong) were chosen during this process.

Participants are allowed to continue with their own usual care (medical and nonmedical) during the study.

#### Participants and Group Allocation

We aim to recruit 594 young women with primary dysmenorrhea. The sample size estimation is based on the comparison of the group receiving the full-featured app (self-care information + self-acupressure) with the group receiving the app version without the self-care information (control intervention II) regarding the primary outcome (NRS after 6 menstrual cycles) that will be treated as a continuous variable. Our previous study showed a mean group difference of 1.4 on the NRS and a standard deviation of 2.15 at the sixth menstrual cycle after the onset of the trial.

Assuming that self-care information has a smaller impact on pain than acupressure, we hypothesize a difference of 0.8 on the NRS between groups. To detect a mean difference of 0.8 point on the NRS after 6 menstrual cycles between the group receiving the full-featured app (with a common standard deviation of 2.15 observed in our previous study) and control intervention II, applying a 2-sided *t* test with a power of 80% and an adjusted alpha of .025, a total of 139 participants will be needed per group (417 women for the 3 arms together). Taking into account a dropout rate of approximately 30% (based on our previous study after 6 cycles), 198 participants per group will be needed (total 594 women).

The eligibility criteria resemble the criteria of our previous study. Women owning an iPhone will be included if they have primary dysmenorrhea, are between the ages of 18 and 34 years, report moderate or severe menstrual pain ≥6 on the NRS; 0=*no pain at all*, 10=*most intense pain imaginable*), and report no existing or planned pregnancy within the next 12 months. During the app-based eligibility screening, the inclusion and exclusion criteria will be assessed by 12 compulsory eligibility questions (Table 2). After the determination of eligibility and obtaining informed consent, participants will be asked to complete the baseline survey before they receive access to the app features depending upon the respective study group allocation. We will use a server-based randomization table created by a statistician using the RANUNI random number generator of the SAS/STAT version 9.2 (SAS Inc) [28]. Participating women will be randomized in a 1:1:1 ratio by block randomization with a fixed block length.

**Table 2:** Eligibility questions.

Eligibility questions	Question type	Criteria
Are you a woman over 18 and below 35 years old?	Yes/no	If no, exclude
Do you suffer from period pain or menstrual cramps during every menstrual cycle?	Yes/no	If no, exclude
Do you suffer from your period pain on more than 5 days outside the period?	Yes/no	If yes, exclude
Do you think your pain started during your teenage years?	Yes/no	If no, exclude
Do you have any prior history of a gynecological disease that is known to be a reason for your period pain?	Yes/no	If yes, exclude
Did you have a period within the last 6 weeks?	Yes/no	If no, exclude
Is your cycle length between 3 and 6 weeks?	Yes/no	If no, exclude
How strong was the most severe pain without medication during your last period?	Numerical on a pain scale from 0 to 10	If <6, exclude
Are you willing to see a doctor when (1) your pain is getting worse than usual, (2) pain medication is not helping, and (3) when you have pain well before or well after the period?	Yes/no	If no, exclude
Are you pregnant?	Yes/no	If yes, exclude
Do you plan to be pregnant within the next 12 months?	Yes/no	If yes, exclude
Is this your iPhone?	Yes/no	If no, exclude and message the user because of data protection, the app should be used only on your own iPhone

#### Efficacy-Effectiveness Continuum

From a methodological point of view, a clinical trial provides more evidence on the effectiveness of an intervention using a pragmatic trial design or on the efficacy side using an explanatory trial design [29,30]. Pragmatic trials are usually considered to study interventions in a real-world setting, whereas explanatory trials are usually designed to investigate interventions in an ideally controlled setting. The PRECIS-2 is a wheel-format tool that helps researchers to consider trial design as more effectiveness or efficacy focused including 9 domains: eligibility criteria, recruitment, setting, organization, flexibility (delivery), flexibility (adherence), follow-up, primary outcome, and primary analysis [31]. The PRECIS-2 scoring system ranges from 1 (most explanatory) to 5 (most pragmatic).

During the design phase of the trial, PRECIS served as a tool to make better informed design decisions [32]. We used the PRECIS-2 tool to assess our app-based trial’s positioning on the pragmatic-explanatory continuum. The authors independently scored the 9 dimensions.

### User Enrollment

The primary recruitment strategy focuses on self-referral through the Apple App Store. On the basis of our experience from the previous trial and the associated stakeholder engagement [12,27], we anticipate that an app-based study for menstrual pain would meet wide acceptance among young women in Germany. Furthermore, we assume that no external advertising (such as posters in public transport or on campus) will be needed for recruitment. A Web-based press release on the Charité university homepage was published on February 28, 2018, (in German and English language), highlighting the results of the previous trial, while also mentioning the new study with the updated app, including a link to the App Store. The media coverage of the app is observed regularly by the study team, using Google search with keywords “selfcare + period pain + Luna,” “selfcare + Luna,” “app + period pain,” “acupressure + period pain” (in German: “Selbsthilfe + Regelschmerzen + Luna,” “Selbsthilfe + Luna,” “app + Regelschmerzen,” “Akupressur + Regelschmerzen”).

The app use will be free of charge; no financial compensation will be provided for participating in the study.

Potential future recruitment strategies will include traditional and Web-based recruitment methods that are also adapted to the respective study sites. These will include information about the ongoing study on printed posters or information leaflets or in social media. In addition, if accepted by the Apple App Store editorial team, we will inform potential users about the study app with the *App of the day* feature option of the Apple App Store for the category *Health and Fitness*.

### User Engagement

When users install and open the study app for the first time, they will be briefly introduced to the study and encouraged to participate. For potential participants who wish to continue, an app-based anonymous eligibility screening and more detailed information about the study will be provided. After the consent process, participants will finish the app-based baseline survey to unlock the intervention interface. This process is based on the onboarding process of Apple’s ResearchKit framework [33]. User flow and conversion rates will be calculated based on the number of downloads, the number of eligible users, and the number of users who finish the baseline survey and enter the study.

In the baseline survey, general information relevant for menstrual pain will be assessed, such as age, education, individual exercise behavior, length of period and level of pain experienced during the period, and use of hormonal contraceptives and pain medications (Table 3). A *skip* button is available for a selection of questions and allows users to skip questions they do not want to answer. User engagement will be measured by usage of *skip* button and baseline survey respondent rate.

**Table 3:** Baseline questions.

Baseline questions and answer field				Skip button	
**Your age**				**X^a^**	
	_____ years				
**BMI calculated from height and weight**				**X**	
	Your height: ____ cm				
	Your body weight: ____ kg				
**What is the highest level of education you have completed so far?**				**X**	
	High school or above				
	Other				
**How long is your cycle usually (the time from the first day of period until the beginning of the next period)?**				**—^b^**	
	_____ days				
**How long is your period usually?**				**—**	
	_____ days				
**What kind of period pain and discomfort do you usually experience? (multi-choice possible)**				**X**	
	Stomach cramps				
	General pain in lower belly				
	Lower back pain				
	Headache				
	Nausea/Vomiting				
	Other symptoms, namely _____				
**Do you use hormonal contraceptives (eg, birth control pills, hormone patch, vaginal ring, or hormonal IUD^c^)?**				**X**	
	No				
	**Yes**				
		**If yes, why do you use hormonal contraceptives?**			
			I use hormonal contraceptives because of my period pain.		
			I use hormonal contraceptives for contraception.		
			I use hormonal contraceptives because of other reasons (for example, acne).		
	If yes, which hormonal contraceptives are you using? ______				
	If yes, how long have you been using hormonal contraceptives? for ____ months and ____ years				
**Have you ever been pregnant?**				**X**	
	No				
	Yes				
	If yes, number of pregnancies:____				
	If yes, number of births:____				
**How intense was the average period pain of the painful days during your last period?**				**—**	
	0 1 2 3 4 5 6 7 8 9 10 (0=no pain at all, 10=most intense pain imaginable)				
**During your last period, how intense was the worst period pain you experienced?**				**—**	
	0 1 2 3 4 5 6 7 8 9 10 (0=no pain at all, 10=most intense pain imaginable)				
**On how many days have you had period pain during your last period?**				**X**	
	_____ days				
**On how many days were you absent from work or education due to period pain during your last period?**				**X**	
	_____ days				
**Have you taken any medication for your period pain?**					
	No				X
	Yes ->if yes, which one: ______				
**Which self-care activities have you done during the previous month because of your period pain? (multi-choice possible)**				**X**	
	No actions				
	Fitness/Gymnastics				
	Jogging/Running				
	Acupressure				
	Yoga				
	Autogenic training				
	Herbal medicine				
	Meditation/Relaxation				
	Homeopathy				
	Local supply of heat				
	Food supplements				
	Tea				
	Others: ______				
**Which self-care activities have you done during the previous month because of other reasons than your period pain? (multi-choice possible)**				**X**	
	No actions				
	Fitness/gymnastics				
	Jogging/running				
	Acupressure				
	Yoga				
	Autogenic training				
	Herbal medicine				
	Meditation/relaxation				
	Homeopathy				
	Local supply of heat				
	Food supplements				
	Tea				
	Others: ______				
**When did you have your last period? Please enter the data of the first day of your last period.**				**—**	
	__.__.____				

^a^X: skip button enabled.

^b^—: skip button disabled.

^c^IUD: intrauterine device.

### Statistical Analysis

The PRECIS-2 score was calculated by summing up the means of each dimension based on the rating results of 11 raters; meanwhile, standard deviations were calculated to show the variability.

For the BCT ratings, the interrater reliability among BCT raters was assessed by intraclass correlations (ICCs) [34,35].

For the assessment of user access, we used the data generated by App Analytics [33] (Apple Inc) to descriptively show the source of product page views and number of downloads.

To assess user engagement, the user conversion rate and the baseline survey response data were calculated using descriptive statistics (frequencies, percentages, means, and standard deviations). The baseline survey variables were extracted from the back-end database and only the missing values of the baseline survey (*skipped* questions) were used for the calculation of the proportion of actually skipped questions among all skippable questions to interpret the user engagement.

All collected data were analyzed with SPSS version 22.0 (SPSS Inc).

### Ethics

The app is prepared for international use and can be currently (October 2019) downloaded in the German App Store and will later be made available in the App Stores of the other participating centers. The study database, the app server, and the primary study center are based in Berlin, Germany. The study was approved by the university’s ethics committee (Charité—Universitätsmedizin Berlin approval Number EA1/364/16). The trial was registered at ClinicalTrials.gov (NCT03432611).

The participation of the study sites in Taichung, Taiwan (approval letter Number CMUH107-REC1-120 by Ethics Committee of China Medical University and Hospital); Sydney, Australia (approval number H13175 by Western Sydney University Human Research Ethics Committee); Florianopolis, Brazil (approval number 3.583.066 by Ethics Committee of Federal University of Santa Catarina), and Baltimore, United States is currently being processed.

## Results

### Study App and Study Design

**Figure 1:**
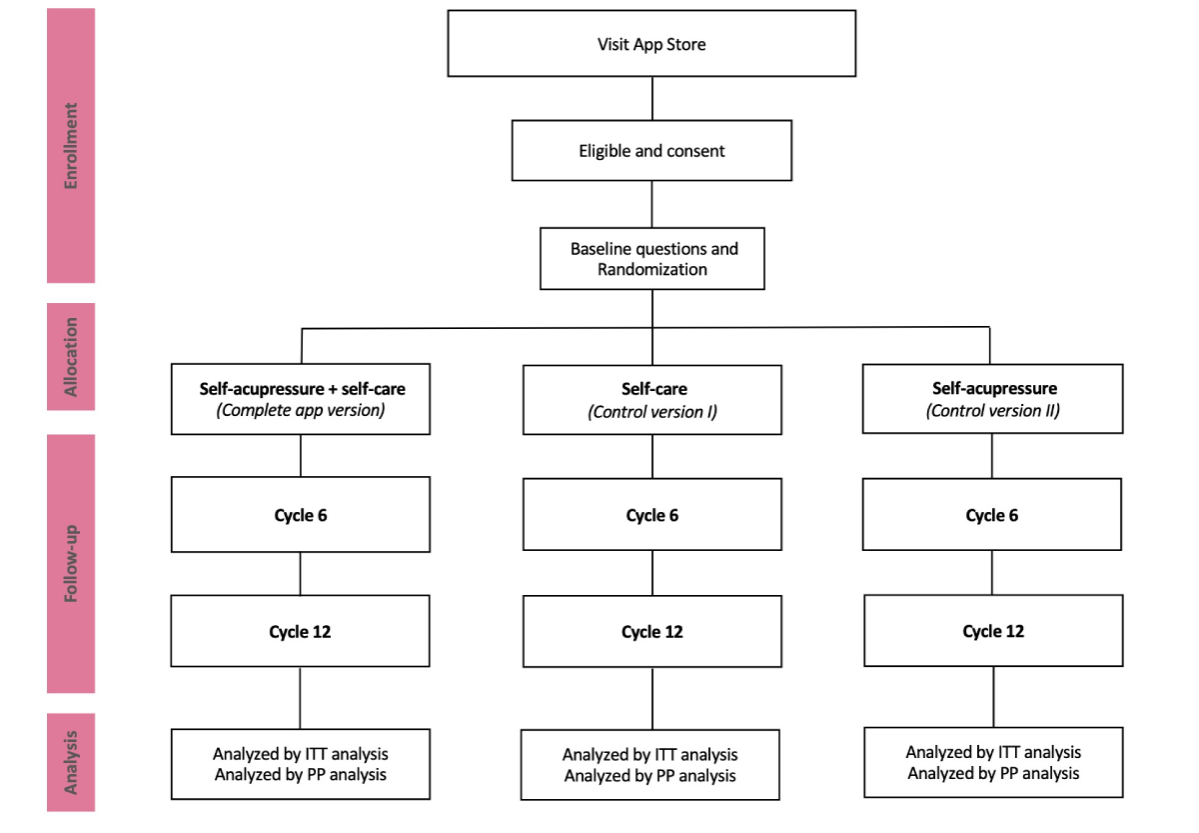
Study design. ITT: intention to treat; PP: per protocol.

The study app is a result of multidisciplinary efforts. The launch of the study app in the App Store will mark the beginning of the fully app-based study: users will be recruited via the Apple App Store, eligibility and consent will be processed by the study app, different self-care interventions will be guided by corresponding app features, and the follow-up will be recorded by app-based survey questions (Figure 1). Detailed screenshots, which depict the user flow in more detail, are listed in Multimedia Appendix 3.

**Figure 2:**
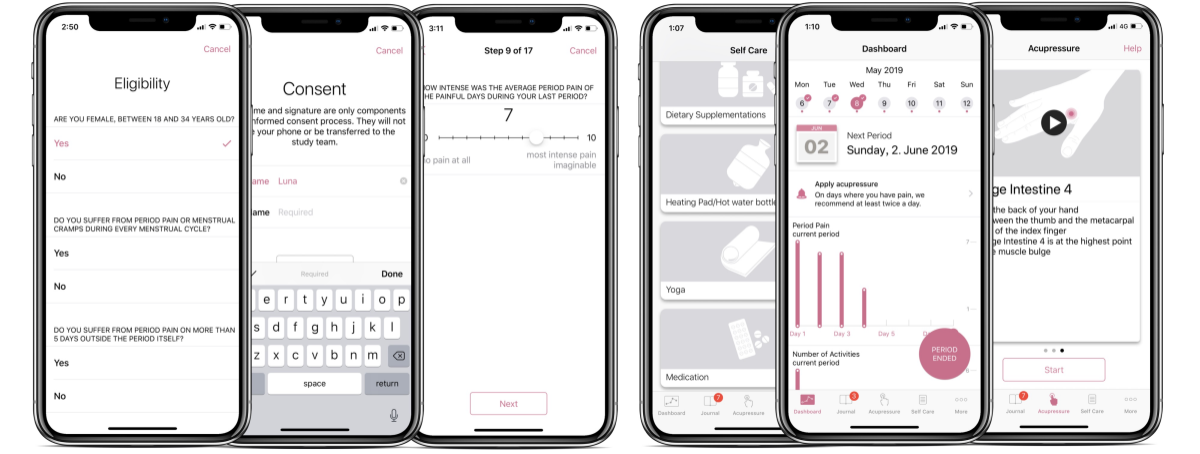
Screenshots of the study app.

The study app will display the intervention components (self-acupressure and self-care information) selectively according to the group allocation. The core features *Dashboard*, *Journal*, and *More* (Figure 2) will be accessible to all users. The *Dashboard* will display feedback according to study progress and answers of survey questions and a prediction of the next period start date. The *Journal* feature will contain a period calendar and an overview of the progress on the survey questions. With the *More* feature, users will be able to set personal identification number lock and notification time. Users’ cycle information, the signed consent form, and a link to the privacy policy will also be displayed there.

#### Behavior Change Technique Ratings

To validate whether the BCTs implemented in the app were properly applied, a developer rating (JW) was compared with ratings of 2 psychologists with BCT expertise (CRP and AR) who had experienced the finalized full-featured app but who had not been part of the app development process. The interrater agreement between the 2 psychologists showed an excellent ICC (ICC=0.954; 95% CI 0.87-0.98). However, the overall interrater agreement including all raters was poor (ICC=0.442; 95% CI 0.07-0.78), that is, the ratings of the BCTs used during the development by the study team, did not correspond well with the ratings of the 2 psychologists. There was no significant difference between ICCs at the item level and the cluster level based on the BCTs taxonomy (v1) [19]. The final agreement that was reached in a consensus meeting is shown in Table 2. Overall, 12 BCTs were identified in the study app. The most frequently implemented BCTs are prompts/cues (5 times), instructions on how to perform behavior (4 times), and demonstration of the behavior (3 times).

#### Efficacy-Effectiveness Continuum Rating

**Figure 3:**
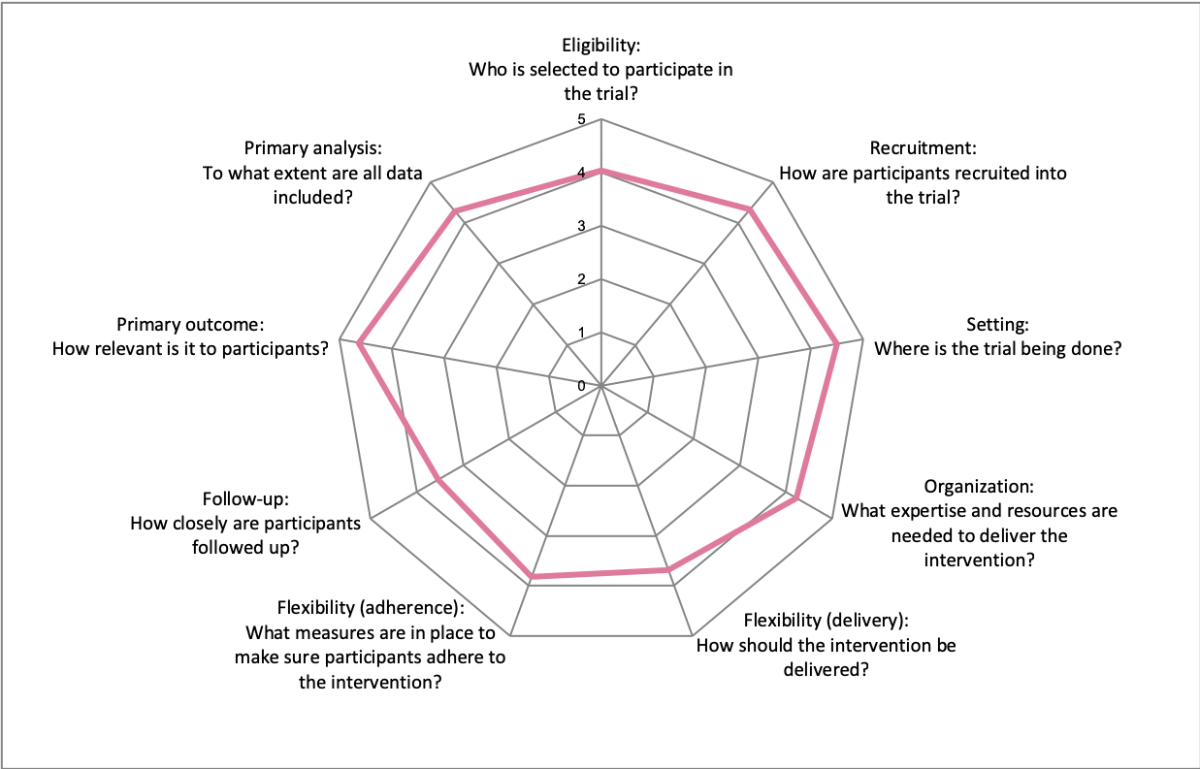
PRECIS-2 rating results of the study design.

On the basis of the rating results of all authors, all 9 dimensions of the PRECIS-2 tool are defined more on the pragmatic side (Figure 3). Thus, this app-based RCT can be considered as a pragmatic trial.

### User Enrollment

**Figure 4:**
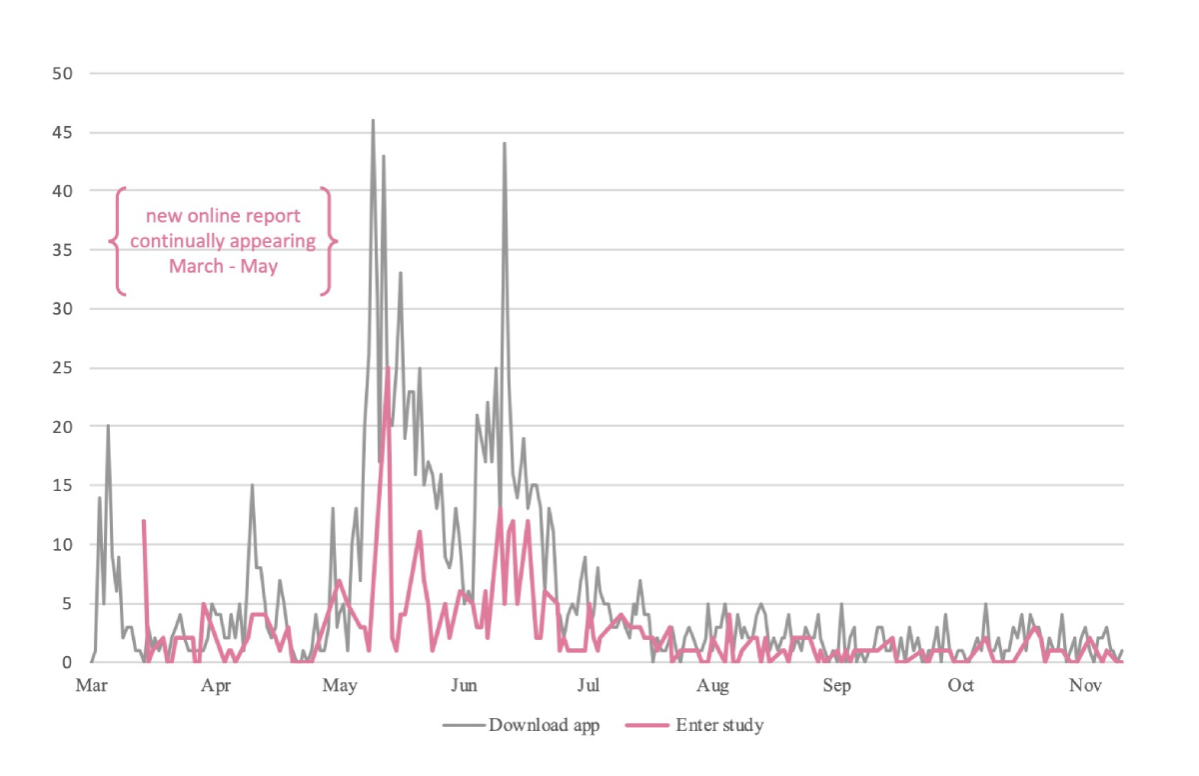
App downloads and new users per day.

Trial recruitment started in February 2018 with the launch of the ResearchKit-based study app in the German App Store. The Web-based press release was well received by the public and the media. By observation of media coverage via Google search during the following 10 weeks, 65 articles or blog entries of pharmacy or health-related websites citing the press releases in English and German could be detected. Overall, 2 printed newspapers reported about this app-based study in German. An increase of media coverage could be observed from March to May 2018. In the weeks following the press release, the app showed continuous increase in both downloads and the number of users (Figure 4).

After 38 weeks in the app store (from February 19, 2018, to November 13, 2018), there were 1458 downloads and 328 users were included into the study (22.5%). On average, we recruited around 8 study participants per week with a peak between May and June after the press release (22 new users per week). Approximately 60% (195/328) of the participants were recruited within these 2 months.

### User Engagement

**Figure 5:**
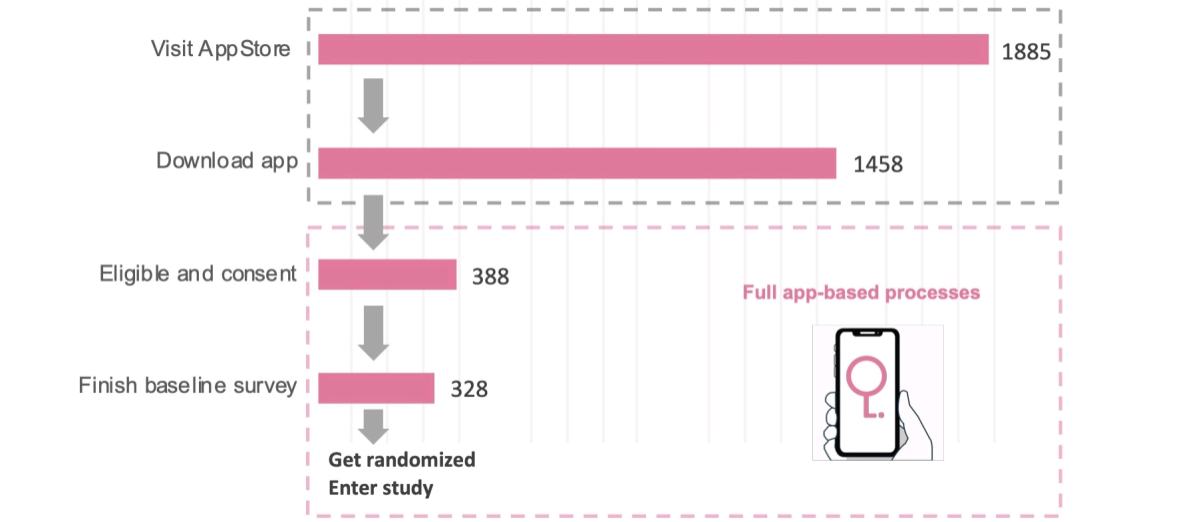
User evolution.

During the first 38 weeks of recruitment, the App Store’s preview of the app was viewed 1885 times. Although 75% of the app’s product page viewers found the app by searching the App Store, 25% found the app by App Store browsing, app referral, or Web referral. The app was downloaded 1458 times. A total of 388 (27%) users passed the 12-question eligibility screening and agreed to consent; 328 of the 388 users (85%) completed the 16-question baseline survey and were recruited to the study. Figure 5 displays the user evolution [14].

For 11 of 16 baseline questions, the *skip* button can be used because these questions are either not related to the primary outcome of the study or their data are not essential for the proper functioning of the app. The usage of the *skip* button of a study sample (328 users) was calculated to evaluate the user engagement in the app-based survey.

Almost all questions of the baseline survey were answered (data completeness of 98.27%; 5157/5248). A total of 276 users (276/328, 84.1%) answered all 16 baseline questions and never used the skip button. Only 3% of the data based on the skippable questions were missing. The question asking for discomfort/symptoms during the period was answered by all users (response rate 100%). For free-text fields, 105 (105/328, 32.0%) of the users provided details about their discomfort/symptoms during their period; 269 (269/328, 82.0%) users provided details about their medication for the question asking about the period pain-related medical history.

## Discussion

### Principal Findings

By using the ResearchKit framework, we successfully developed a study app for a fully app-based pragmatic RCT for young women with primary dysmenorrhea. The app is easily accessible via self-referral and can be used as a self-care and study tool for a highly relevant condition. The available data already indicate a high level of user engagement with the study app. We also realized that the early involvement of behavioral science experts is of great importance for the development of app-based trials.

In a young population that widely uses smartphones, a digital intervention, such as the study app, provides low entry barriers. It offers easy access to evidence-based self-care information for menstrual pain and tools to improve healthy behavior. We believe that recruitment is not only influenced by the app itself but also by the way of communicating the study. We observed a substantial increase in recruitment rates following the publication of a press release on our university’s websites and corresponding media coverage. A causal relationship in the recruitment increase seems to be very probable. After 5 months without actively communicating the study with media or information material, we still could observe a basic recruitment of about 1 new study participant per day.

Almost all research or self-care apps include BCT elements, such as prompts/cues to fill in questionnaires (self-monitoring) or to engage in app- specific intervention components. Dialog boxes are also used to give feedback on behavior or to promote self-belief [19]. However, the adequate implementation and the proper description of the applied BCTs are not easy to achieve. Therefore, it is important to involve psychologists or behavioral scientists in the design and development of an app [36,37]. The review of the use of the BCT taxonomy during the development of the trial revealed some discrepancies between the study team members and the psychologists that were involved in the ratings. For future studies, the behavior change wheel framework by Michie et al [38] will be applied before the app development to improve the design and implementation of app-based interventions [39]. Moreover, the mechanisms and efficacy of BCTs implemented need to be further explored in mHealth research settings.

As in our previous mHealth studies, the app and trial simultaneously shaped each other during the trial design and app development process. In conventional RCTs, the trial intervention and outcomes are usually very standardized as they are described in the study protocol. However, during the development and coding process of the study app, we regularly made adaptations of the study protocol because of technical and design aspects. For example, during the development process, we realized that the digitally collected data can be used to give the users an overview of study progress and symptom improvement that subsequently became part of the intervention strategy. Branching within a question (the answer of an item impacts the next question choices) and combining different question types were not possible with the standard ResearchKit framework. Moreover, baseline questions had to be limited to reduce the time spent until finalization of onboarding, that is, the whole process from introduction, eligibility screening, and participant consent until completion of baseline survey and the random allocation to the respective intervention group. However, the final onboarding process in our research app was longer than what users of consumer apps might usually accept. This could have resulted in a loss of potential study participants. Some baseline questions typical for research studies, such as questions about partnership and income, were omitted because of privacy concerns. It was not necessary to collect body weight and height as PII data, as they were only used for BMI calculation on the user’s iPhone and not transferred to the study backend. The study design also impacted some technical decisions. For instance, to limit recall bias, questions that required daily answers before and during the period will expire after 7 days. Moreover, the way symptoms are measured or tracked in an app is limited to validated and commonly used outcomes. NRS or Likert scales are used instead of more consumer-oriented approaches, such as individualized icons or emojis to record mood or pain. This might limit the user experience.

### Limitations

In addition to the limitation of the development process already described above, several other related limitations have to be taken into account. The decision to focus on Apple’s iOS only enabled the use of Apple ResearchKit and avoided the difficulties associated with developing for 2 operating systems simultaneously, as was done in our previous trials [12,27]. Owing to this decision, only women using an iPhone can participate in the study, which consequently might introduce selection bias and therefore limit the generalizability of the study. Moreover, the impact of technical updates (ie, ResearchKit and iOS updates) or other potential adaptations of the app during the course of the study is not clear yet, but these adaptations will be thoroughly reported in the results paper of the trial.

Our study is also subject to some limitations from the access perspective. The numbers of App Store’s visitors and downloads are generated by Apple’s App Analytics, which we do not control. This is the only source to estimate the number of subjects interested in our study because of our anonymous study design. However, we think that it is important to also include App Analytics’ data despite its nonstudy purpose. Taking advantage of these resources from the mHealth ecosystem might help future app-based studies. To be eligible to use our study app, individuals who downloaded the app had to pass our 12-question eligibility screening that is based on our relatively strict inclusion and exclusion criteria. However, for the assessment of user evolution, we could only record the number of eligible users who gave consent because of ResearchKit’s design restrictions and our privacy rules. As a result, we lack knowledge about the reasons for ineligibility. In addition, although the participant’s eligibility and survey data underwent comparably strict plausibility checks that we have implemented in the app, fake users and fraud registration for the study cannot be completely ruled out. However, our fully remote study allows user behavior in a real-life setting [12,21,40]. Additional plausibility checks will be developed before the analysis of the results. Another way to access the app could be on recommendation of a gynecologist and/or family physician within a therapeutic setting. Further study is required to make a definite conclusion about the extent to which the app might be of use in such a setting.

Data on user engagement in our study are limited so far. The only indicator we currently use for assessing engagement is based on the completion and response of the baseline questions. Commercial apps often use analytic tools to track user interaction with the app. These data can be used for the evaluation of engagement [41], the optimization of the app, or the addition of new features. In a study setting with strict privacy considerations, we do not use these tools. In addition, adherence would be a good measurement for user engagement. Data about adherence is not available yet but will be considered as an outcome of the study.

### Comparison With Prior Studies

The study app and the app-based trial result from adaptation and amendments of our previous *AKUD* trial [12,27]. The inclusion criteria of the research population are based on the previous trial but were modified to meet the necessities of remote recruitment. In the previous *AKUD* trial, participants were recruited through onsite recruitment by 1 study center in Berlin, Germany facilitated by advertisements (posters, flyers, leaflets, students email lists, and subway advertisements). Baseline data were recorded with paper-and-pencil surveys. This way, it took 20 months to reach the recruitment target of 221 participants [12,27]. With the ResearchKit-based study app, we are now able to reach participants across Germany.

For the assessment of access of app studies, Anguera et al [40] reported the recruitment number, whereas Zens et al [14] reported the consent/download rate. The percentage of consented participants (27%) in our trial is lower than in other ResearchKit studies [14,42,43]. The mPower study [42] reported 35% consent/download rate, whereas the *Back on Track* study [14] reported 58%. The differences might be explained by the observational character of these studies and the application of a comparably strict eligibility process in our study with 12 eligibility questions.

User adherence and survey response rate are usually considered to be the measurements for evaluating engagement in app studies [40,44,45]. However, as adherence data of the current trial are not available in the current stage of the study, the baseline survey response is used as a proxy for engagement.

The ResearchKit framework has been used for studies for many health conditions, such as asthma [43], acute anterior cruciate ligament ruptures [14], and Parkinson disease [42] since its launch in 2015 [46]. To our knowledge, no ResearchKit clinical trial for pain conditions has been conducted yet. Thurnheer et al [5] reported 15 studies without ResearchKit and their efficacy in a systematic review of app-based studies for pain management. App-based studies have been conducted both for acute pain such as acute needle stick pain [47] and acute pain before coronarography [48] and for chronic pain such as chronic cancer pain [49], neck pain [50], and low back pain [24,51]. No mHealth-based interventional trial has been conducted for examining the influence of an app promoting health behavior and the use of acupressure on menstrual pain [5,52] so far. Regarding the high prevalence of menstrual pain and the increasing ownership of smartphones [12,53,54], our trial might provide data that can have practical public health implications.

### Conclusions

Conducting an evidence-based and up-to-date app study requires multidisciplinary efforts. The resulting ResearchKit-based study app for menstrual pain is accessible for the target population with positive user engagement. However, future research is necessary to investigate the determinants of user engagement, optimal BCT application, and potential clinical scenarios for app use.

## Appendix

Multimedia Appendix 1 Self care feature.

Multimedia Appendix 2 Acupressure feature.

Multimedia Appendix 3 Screenshots and user flow to enter the study.

Multimedia Appendix 4 CONSORT-EHEALTH checklist (V 1.6.1).

## Abbreviations

BCT: behavior change technique
ICC: intraclass correlation
mHealth: mobile health
NICM: National Institute of Complementary Medicine
NRS: numerical rating scale
PII: personally identifiable information
RCT: randomized controlled trial
