# Supplementary material for: International ResearchKit App for Women with Menstrual Pain: Development, Access, and Engagement
Source: JMIR Mhealth Uhealth. 2020 Feb 11;8(2):e14661. doi: 10.2196/14661 (PMC7055820; doi:10.2196/14661)
Supplement: Multimedia Appendix 1 [file mhealth_v8i2e14661_app1.pdf]

Exercises

Exercises seems to reduce menstrual symptoms such as pain in some studies. Exercising has a number of benefits, therefore exercise is a good place to start when looking to treat menstrual pain.

There are various kinds of exercises you can do, i.e. full-body exercise like cardio or muscle training. Some women benefit from exercising and it helps reduce their menstrual pain, while other women find it better to rest. In case you aren't sure if exercising will help you, try doing some gentle exercise such as walking to see if it makes you feel better.

1. *Roger PS, Andrew MK. Patient education: Painful menstrual periods (dysmenorrhea) (Beyond the Basics). In: UpToDate Available at: http://www.uptodate.com/contents/painful-menstrual-periods-dysmenorrhea-beyond-the-basics (Accessed September 9, 2017)*
2. *Banikarim C, Chacko MR, Kelder SH. Prevalence and impact of dysmenorrhea on Hispanic female adolescents. Arch Pediatr Adolesc Med. 2000;154(12):1226-9.*
3. *Golub LJ, Menduke H, Lang WR. Exercise and dysmenorrhea in young teenagers: A 3-year study. Obstet Gynecol. 1968;32(4):508-11.*

Dietary Supplementations

A variety of dietary and vitamin therapies have been studied for the relief of dysmenorrhea. For example, increasing your intake of Omega-3 can help reduce menstrual pain. Omega-3 fatty acids are found in fish, walnuts and dark leafy green vegetables.

If you can't add these to your diet, you can try using an Omega-3 supplement such as fish oil, krill oil or flaxseed oil or vitamin supplements.

However, the effectiveness of fenugreek, ginger, valerian, zataria, zinc sulphate, fish oil and vitamin B1 for dysmenorrhea seem to be limited.

1. *Proctor ML, Murphy PA. Herbal and dietary therapies for primary and secondary dysmenorrhoea. Cochrane Database Syst Rev. 2001;(3):CD002124.*
2. *Pattanittum P, Kunyanone N, Brown J, et al. Dietary supplements for dysmenorrhoea. Cochrane Database Syst Rev. 2016;3:CD002124.*

Heating Pad/Hot water bottle

Applying heat to the lower abdomen with a heating pad, hot water bottle or self-heating patch can significantly reduce pain, often as well as treatment with an NSAID. It is

important to avoid skin burns with a heating pad or hot water bottle that is too hot; a temperature of approximately 104ºF (40ºC) is recommended. The heat can be applied as often as it is needed. Using heat in addition to ibuprofen may speed the relief of pain.

1. *Akin MD, Weingand KW, Hengehold DA, Goodale MB, Hinkle RT, Smith RP. Continuous low-level topical heat in the treatment of dysmenorrhea. Obstet Gynecol. 2001;97(3):343-9.*

Yoga

There is some evidence that yoga is effective in reducing painful menstruation. However, further study is needed to confirm this. Please consult your gynecologist before practicing yoga against menstruation pain, especially when you are at the beginner level of yoga.

1. *Roger PS, Andrew MK. Patient education: Painful menstrual periods (dysmenorrhea) (Beyond the Basics). In: UpToDate Available at: http://www.uptodate.com/contents/painful-menstrual-periods-dysmenorrhea-beyond-the-basics (Accessed September 9, 2017)*

Medication

Doctors usually prescribe NASIDs for dysmenorrhea. Please follow the instructions, and please take analgesic meditation prescribed by the doctor although some are available without a prescription.

You should not use our recommendation instead of seeing a gynecologist. Only medical check-ups at the gynecologist will be able to detect severe diseases. We recommend to see your gynecologist regularly.

Please contact a physician especially when:

- The pain is getting worse
- Pain medicine isn't helping
- You also have pain well before or well after your period

1. *Campbell MA, Mcgrath PJ. Use of medication by adolescents for the management of menstrual discomfort. Arch Pediatr Adolesc Med. 1997;151(9):905-13.*
2. *Roger PS, Andrew MK. Patient education: Painful menstrual periods (dysmenorrhea) (Beyond the Basics). In: UpToDate Available at: http://www.uptodate.com/contents/painful-menstrual-periods-dysmenorrhea-beyond-the-basics (Accessed September 9, 2017)*
