# Supplementary material for: International ResearchKit App for Women with Menstrual Pain: Development, Access, and Engagement
Source: JMIR Mhealth Uhealth. 2020 Feb 11;8(2):e14661. doi: 10.2196/14661 (PMC7055820; doi:10.2196/14661)
Supplement: Multimedia Appendix 2 [file mhealth_v8i2e14661_app2.pdf]

When to Apply Acupressure

Apply acupressure throughout the 5 days leading up to your period until the last day of your period, twice a day if possible.
On days where you have pain during your period, we recommend applying acupressure at least twice a day, up to 5 times a day if you like.

How to Apply Acupressure

First, find a comfortable sitting position. Using your thumb, massage each acupressure point in small circles with medium pressure (firmly, but not so that you injure yourself). Focus on the acupressure point as you are massaging it. Make sure that you use circular movements and do not rub back and forth. While massaging, you should notice a distinct sensation, e.g. tingling, warmth, heaviness or aching. Massage each of the three points one after the other for one minute, on both sides of your body. We suggest to use the timer of the app.

Spleen 6

- On the inside of your leg
- Four fingers above the highest point of the inner ankle bone
- The acupressure point is in a depression just behind the tibia

Liver 3

- On the top of the foot
- Move your finger or thumb upwards between your big toe and the second toe
- The acupressure point is in a depression just before the metatarsal bones meet

Large Intestine 4

- On the back of your hand
- Between the thumb and the metacarpal bone of the index finger
- Large Intestine 4 is at the highest point of the muscle bulge

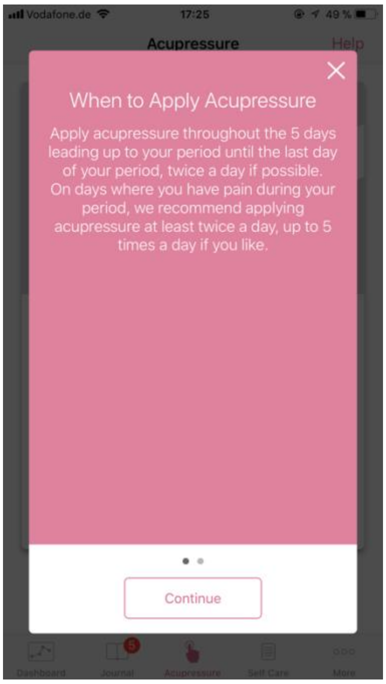
Vodafone.de 17:25 49 %
Acupressure
Help
When to Apply Acupressure
Apply acupressure throughout the 5 days leading up to your period until the last day of your period, twice a day if possible. On days where you have pain during your period, we recommend applying acupressure at least twice a day, up to 5 times a day if you like.
Continue

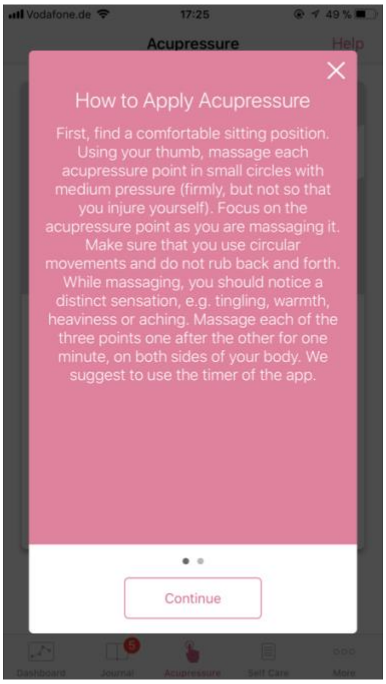
Vodafone.de 17:25 49 %
Acupressure
Help
How to Apply Acupressure
First, find a comfortable sitting position. Using your thumb, massage each acupressure point in small circles with medium pressure (firmly, but not so that you injure yourself). Focus on the acupressure point as you are massaging it. Make sure that you use circular movements and do not rub back and forth. While massaging, you should notice a distinct sensation, e.g. tingling, warmth, heaviness or aching. Massage each of the three points one after the other for one minute, on both sides of your body. We suggest to use the timer of the app.
Continue

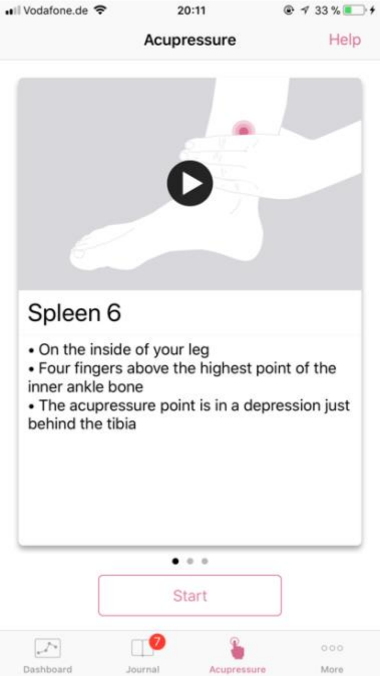
Vodafone.de 20:11 33 %
Acupressure
Help
Spleen 6
• On the inside of your leg
• Four fingers above the highest point of the inner ankle bone
• The acupressure point is in a depression just behind the tibia
Start
Dashboard
Journal
Acupressure
More

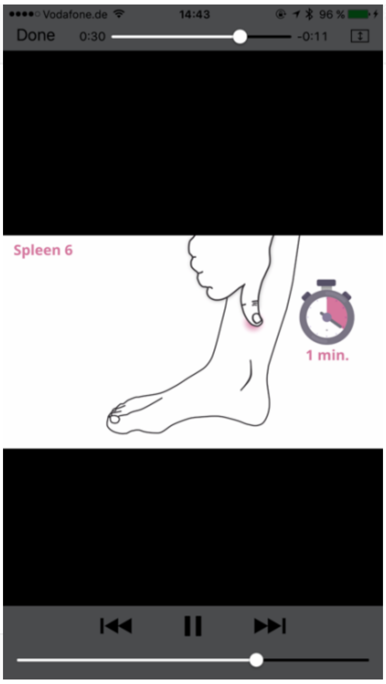
Vodafone.de 14:43 96 %
Done
0:30
-0:11
Spleen 6
1 min.

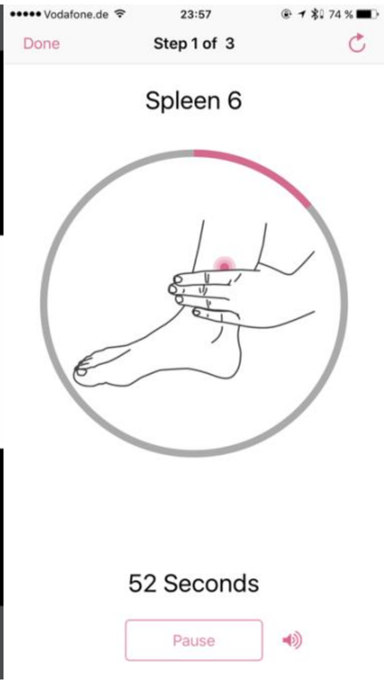
Vodafone.de 23:57 74 %
Done
Step 1 of 3
Spleen 6
52 Seconds
Pause
