# Supplementary figures and images for: International ResearchKit App for Women with Menstrual Pain: Development, Access, and Engagement
Source: JMIR Mhealth Uhealth. 2020 Feb 11;8(2):e14661. doi: 10.2196/14661 (PMC7055820; doi:10.2196/14661)

Download from App Store

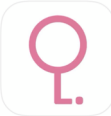

Luna. - Selfcare

Active against period pain

OPEN

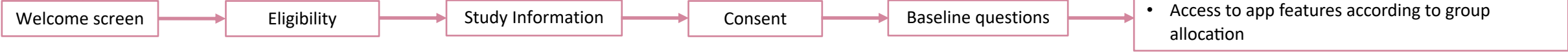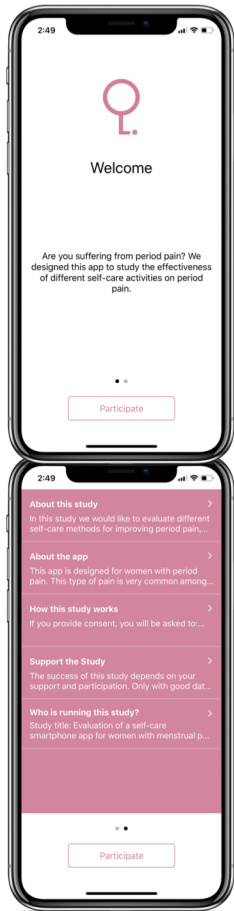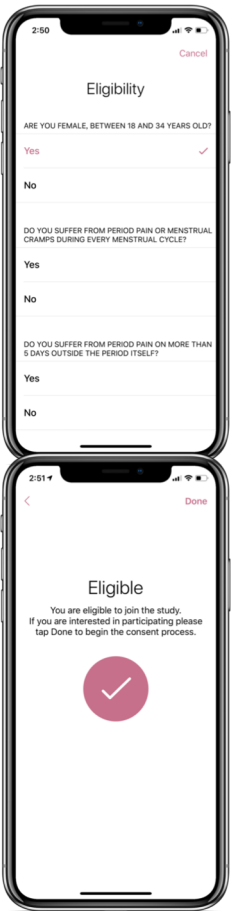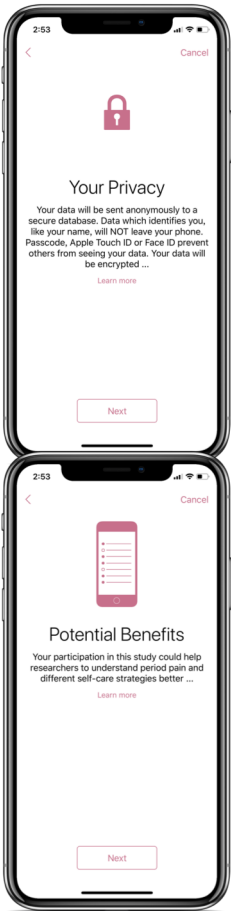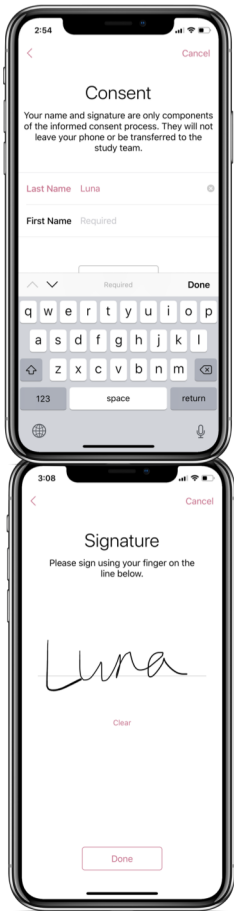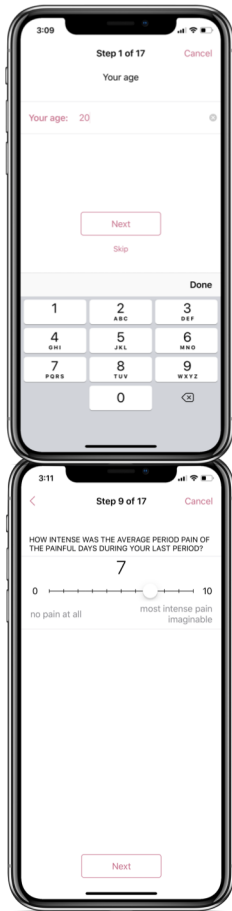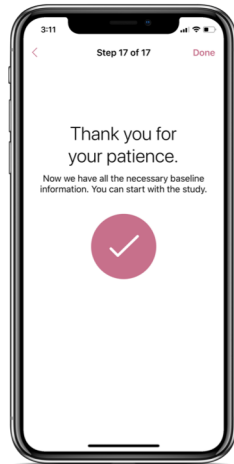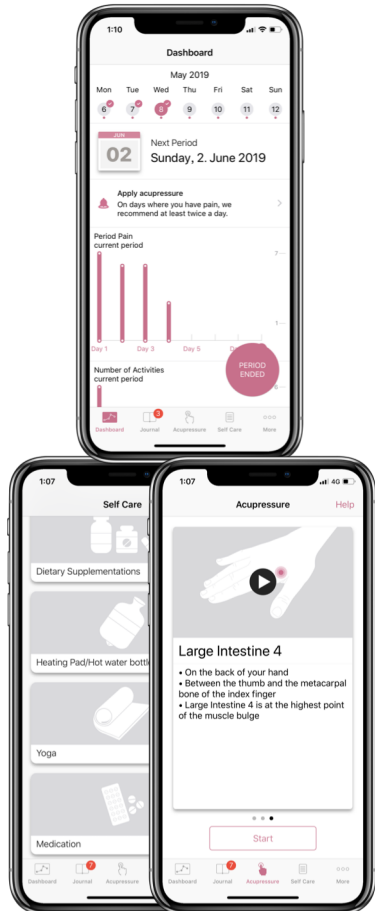

Supplement: Multimedia Appendix 3 [file mhealth_v8i2e14661_app3.pdf]
